# Enhanced late blight resistance by engineering an EpiC2B‐insensitive immune protease

**DOI:** 10.1111/pbi.14209

**Published:** 2023-10-30

**Authors:** Mariana Schuster, Sophie Eisele, Liz Armas‐Egas, Till Kessenbrock, Jiorgos Kourelis, Markus Kaiser, Renier A. L. van der Hoorn

**Affiliations:** ^1^ Plant Chemetics Laboratory, Department of Biology University of Oxford Oxford UK; ^2^ Chemische Biologie, Zentrum für Medizinische Biotechnologie, Fakultät für Biologie Universität Duisburg‐Essen Essen Germany; ^3^ Present address: Leibniz Institute of Plant Biochemistry Halle Germany; ^4^ Present address: Department of Biosystems Science and Engineering ETH Zürich Basel Switzerland; ^5^ Present address: The Sainsbury Laboratory University of East Anglia, Norwich Research Park Norwich UK

**Keywords:** protease, engineering, inhibitor, disease resistance, crop protection

Papain‐like immune proteases (PLCPs) are promising engineering targets for crop protection, given their significant roles in plant immunity for key crops such as tomato, maize and citrus (Misas‐Villamil *et al*., 2016). The wide range of pathogen‐secreted PLCP inhibitors highlights the importance of these proteases in defending against various pathogens. Depletion of the apoplastic immune PLCP *Phytophthora*‐inhibited protease 1 (Pip1) from tomato, for instance, causes hyper‐susceptibility to bacterial, fungal and oomycete tomato pathogens (Ilyas *et al*., 2015). Immunity by Pip1 in wild‐type tomato is, however, suboptimal since Pip1 is suppressed during infection by diverse pathogen‐secreted inhibitors, such as the cystatin‐like EpiC2B from the oomycete late blight pathogen *Phytophthora infestans* (Tian *et al*., 2007). Here, we tested whether we could increase Pip1‐based immunity against late blight by engineering Pip1 into an EpiC2B‐insensitive protease. To guide Pip1 mutagenesis, we generated a structural model of the EpiC2B‐Pip1 complex using AlphaFold‐Multimer (Evans *et al*., 2022). This structural model represents a classic interaction between the tripartite wedge of cystatin (EpiC2B) in the substrate binding groove of papain (Pip1). This model indicated that engineering Pip1 to prevent inhibition without affecting Pip1 substrate specificity is possible because the interaction surface of Pip1 with EpiC2B is larger than the substrate binding groove (Figure 1a).

**Figure 1:**
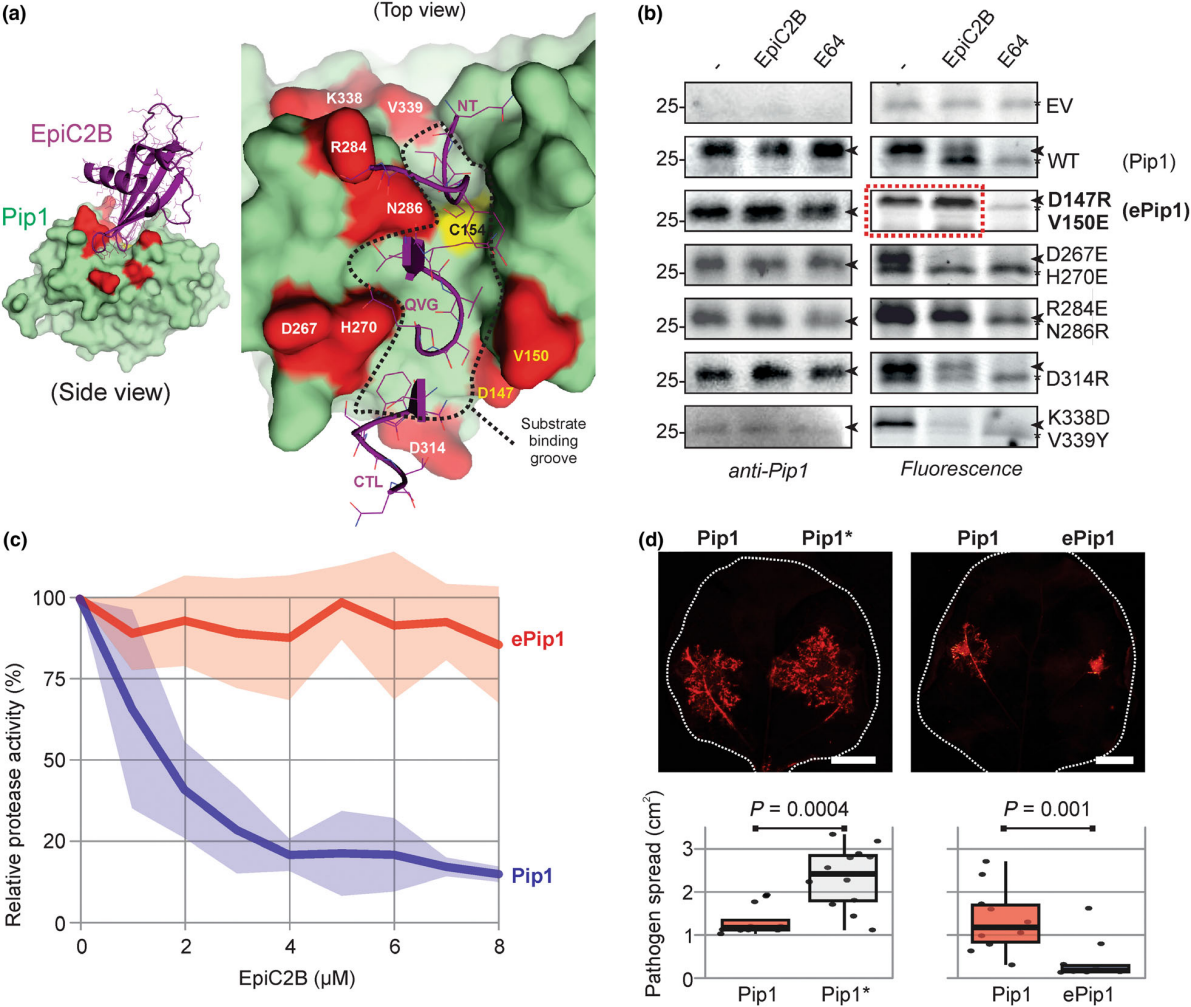
Engineered, EpiC2B‐insensitive Pip1 reduces the growth of *P. infestans* on *N. benthamiana*. (a) Structural model of the Pip1‐EpiC2B complex generated with AlphaFold‐Multimer (Evans *et al*., 2022) showing the substrate binding groove (dashed line), and the residues selected for mutagenesis in Pip1 (red). The right zoomed image shows the tripartite loops of EpiC2B interacting with the substrate binding groove: the N terminus (NT), the middle loop containing the QxVxG (QVG) motif and the C‐terminal loop (CTL) containing a conserved tryptophane. (b) Pip1 mutants are active proteases with altered sensitivities to EpiC2B inhibition. Apoplastic fluids isolated from agroinfiltrated leaves transiently expressing (mutant) Pip1 were preincubated for 30 min with and without 100 μM E‐64 or 3 μM EpiC2B, and then labelled for 3 h with 0.2 μM TK011. Samples were separated on SDS‐PAGE gels, scanned for fluorescence and analysed by α‐Pip1 Western blot. Original, uncropped images are shown in Figure S2. (c) Two residue substitutions render Pip1 insensitive to EpiC2B. Apoplastic fluids isolated from agroinfiltrated leaves transiently expressing Pip1 and engineered ePip1 were preincubated for 30 min with and without 100 μM E‐64, or increasing concentrations of EpiC2B, and then labelled for 3 h with 0.2 μM TK011. Samples were separated on SDS‐PAGE gels and scanned for fluorescence. Signal intensity was quantified. Values are depicted as a percentage of the labelling detected without inhibitor. Confidence zones represent SE of *n* = 3 replicates. (d) Engineered Pip1 restricts *P. infestans* growth in *N. benthamiana*. *N. benthamiana* leaf halves were agroinfiltrated with wild‐type Pip1, catalytically dead Pip1* or engineered ePip1 and 3 days later, the leaves were detached, and zoospores of *P. infestans* strain 88069 tD were drop‐inoculated onto each leaf half. Leaves were incubated for further 7–9 days and imaged for fluorescence. The area containing fluorescent hyphae was measured. *P* values correspond to a paired *t*‐test of *n* > 8 replicates. This experiment was repeated twice with similar results (see Figure S3).

We selected nine residues for targeted mutagenesis of Pip1 that are predicted to directly interact with EpiC2B but are not in the substrate binding groove (Figure 1a). To generate the greatest disruption in protease‐inhibitor interaction, we substituted these residues into bulky amino acids of the opposite charge. Following this strategy, we generated four double mutants and one single mutant of Pip1 and produced these proteins *in planta* via agroinfiltration (Table S1, S2). All proteins were detected in the apoplast of agroinfiltrated *Nicotiana benthamiana* plants using the anti‐Pip1 antibody (Figure 1b), and all are active proteases because they reacted with TK011, a new fluorescent activity‐based probe for PLCPs (Figure S1). Preincubation with 3 μM purified EpiC2B reduced labelling of Pip1 and some of the Pip1 mutants, but the D147R/V150E mutant of Pip1 seems insensitive to EpiC2B inhibition (Figure 1b) and was hence called engineered Pip1 (ePip1). Preincubation with increasing EpiC2B concentrations revealed that ePip1 is insensitive to up to 8 μM EpiC2B, whereas wild‐type Pip1 can be inhibited by 1 μM EpiC2B (Figure 1c).

We next tested whether ePip1 enhances immunity against late blight. Taking advantage of the fact that *N. benthamiana* is a natural null mutant for Pip1 (Kourelis *et al*., 2020) and that infections of agroinfiltrated leaves are well‐established assays for *P. infestans*, we first tested whether transient expression of wild‐type Pip1 enhances late blight immunity. Wild‐type Pip1 and the catalytic mutant of (C153A/C154A, Pip1*) were transiently expressed side‐by‐side in *N. benthamiana* leaves, and the agroinfiltrated area was infected with zoospores of transgenic *P. infestans* expressing fluorescent tDtomato (Chaparro‐Garcia *et al*., 2011). Tissue expressing Pip1 supported reduced *P. infestans* growth when compared to the Pip1* negative control (Figure 1d), confirming that Pip1 is an immune protease, also when expressed in *N. benthamiana*. Importantly, growth of fluorescent *P. infestans* was further reduced in tissues expressing ePip1 when compared to wild‐type Pip1 (Figure 1d), demonstrating that EpiC2B‐insensitive ePip1 increases immunity against *P. infestans*.

Thus, we engineered the Pip1 immune protease from tomato to render it insensitive to protease‐inhibitor EpiC2B from *P. infestans* by mutating two residues. These two residues are invariant in Pip1 in both tomato (46 genomes, Li *et al*., 2023; Zhou *et al*., 2022) and potato (104 genomes, Tang *et al*., 2022), and these substitutions can be introduced into tomato cultivars by genome editing. In the absence of known Pip1 substrates, we cannot exclude that some of this immunity might be caused by altered substrate specificity, but this is unlikely given the location of these two residues outside the substrate binding groove (Figure 1a). Durability of this immunity can be increased by introducing additional substitutions that suppress EpiC2B inhibition, or by building a multigene array that encodes different ePip1 variants. Similar structure‐guided mutagenesis can be performed on Pip1 to increase immunity against other pathogens and on other secreted immune proteases to increase immunity in other crops. This approach enhances the natural extracellular immunity in crops that can be achieved using genome editing and offers a distinct alternative to engineering pathogen perception mechanisms.

## Conflict of interest

None declared.

## Author contributions

MS designed and performed most of the experiments with the help of LA and SE. LA generated most Pip1 mutant strains. JK generated pJK157 and pJK489. TK and MK synthetized TK011. RvdH conceived and supervised the project. MS and RvdH wrote the article, with input from all authors.

## Supporting information


**Figure S1** TK011 is an E‐64‐based fluorescent probe.
**Figure S2** Uncropped gel and blot used for Figure 1B.
**Figure S3** Overexpression of ePip1 in *N. benthamiana* restricts *P. infestans* spread.

**Table S1** Plasmids used in this study.
**Table S2** Primers and oligos used in this study.

## Data Availability

All the data are presented as supplemental figures.

## References

[pbi14209-bib-0001] Chaparro‐Garcia, A. , Wilkinson, R.C. , Gimenez‐Ibanez, S. , Findlay, K. , Coffey, M.D. , Zipfel, C. , Rathjen, J.P. *et al*. (2011) The receptor‐like kinase SERK3/BAK1 is required for basal resistance against the late blight pathogen *Phytophthora infestans* in *Nicotiana benthamiana* . PLoS One, 6, e16608. 21304602 10.1371/journal.pone.0016608 PMC3029390

[pbi14209-bib-0002] Evans, R. , O'Neill, M. , Pritzel, A. , Antropova, N. , Senior, A. , Green, T. , Žídek, A. *et al*. (2022) Protein complex prediction with AlphaFold‐Multimer. *bioRxiv*, 2021.2010.2004.463034.

[pbi14209-bib-0003] Ilyas, M. , Hörger, A.C. , Bozkurt, T.O. , van den Burg, H.A. , Kaschani, F. , Kaiser, M. , Belhaj, K. *et al*. (2015) Functional divergence of two secreted immune proteases of tomato. Curr. Biol. 25, 2300–2306. 26299516 10.1016/j.cub.2015.07.030

[pbi14209-bib-0004] Kourelis, J. , Malik, S. , Mattinson, O. , Krauter, S. , Kahlon, P.S. , Paulus, J.K. and van der Hoorn, R.A.L. (2020) Evolution of a guarded decoy protease and its receptor in solanaceous plants. Nat Commun. 11, 4393. 32879321 10.1038/s41467-020-18069-5 PMC7468133

[pbi14209-bib-0005] Li, N. , He, Q. , Wang, J. , Wang, B. , Zhao, J. , Huang, S. , Yang, T. *et al*. (2023) Super‐pangenome analyses highlight genomic diversity and structural variation across wild and cultivated tomato species. Nat. Genet. 55, 852–860. 37024581 10.1038/s41588-023-01340-y PMC10181942

[pbi14209-bib-0006] Misas‐Villamil, J.C. , van der Hoorn, R.A.L. and Doehlemann, G. (2016) Papain‐like cysteine proteases as hubs in plant immunity. New Phytol. 212, 902–907. 27488095 10.1111/nph.14117

[pbi14209-bib-0007] Tang, D. , Jia, Y. , Zhang, J. , Li, H. , Cheng, L. , Wang, P. , Bao, Z. *et al*. (2022) Genome evolution and diversity of wild and cultivated potatoes. Nature, 606, 535–541. 35676481 10.1038/s41586-022-04822-x PMC9200641

[pbi14209-bib-0008] Tian, M. , Win, J. , Song, J. , van der Hoorn, R. , van der Knaap, E. and Kamoun, S. (2007) A *Phytophthora infestans* cystatin‐like protein targets a novel tomato papain‐like apoplastic protease. Plant Physiol. 143, 364–377. 17085509 10.1104/pp.106.090050 PMC1761951

[pbi14209-bib-0009] Zhou, Y. , Zhang, Z. , Bao, Z. , Li, H. , Lyu, Y. , Zan, Y. , Wu, Y. *et al*. (2022) Graph pangenome captures missing heritability and empowers tomato breeding. Nature, 606, 527–534. 35676474 10.1038/s41586-022-04808-9 PMC9200638

