# Supplementary material for: Enhanced late blight resistance by engineering an EpiC2B‐insensitive immune protease
Source: Plant Biotechnol J. 2023 Oct 30;22(2):284–6. doi: 10.1111/pbi.14209 (PMC10826977; doi:10.1111/pbi.14209)
Supplement: Supplementary file 1 — Figure S1 TK011 is an E‐64‐based fluorescent probe. Figure S2 Uncropped gel and blot used for Figure 1B. Figure S3 Overexpression of ePip1 in N. benthamiana restricts P. infestans spread. [file PBI-22-284-s001.pdf]

Supplemental figures

Enhanced late blight resistance by engineering an EpiC2B-insensitive immune protease Mariana Schuster, Sophie Eisele, Liz Armas-Egas, Till Kessenbrock, Jiorgos Kourelis, Markus Kaiser, and Renier A. L. van der Hoorn

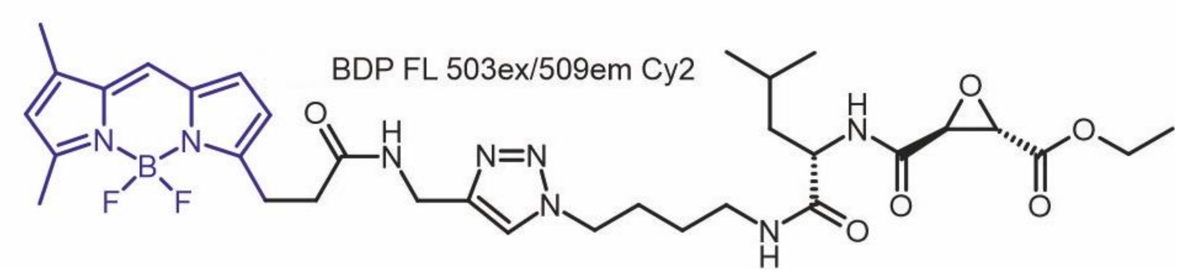

Figure S1 TK011 is an E-64-based fluorescent probe.
Structure of the TK011 probe consisting of a E64-azide linked to an BDP-FL-alkyne (blue).

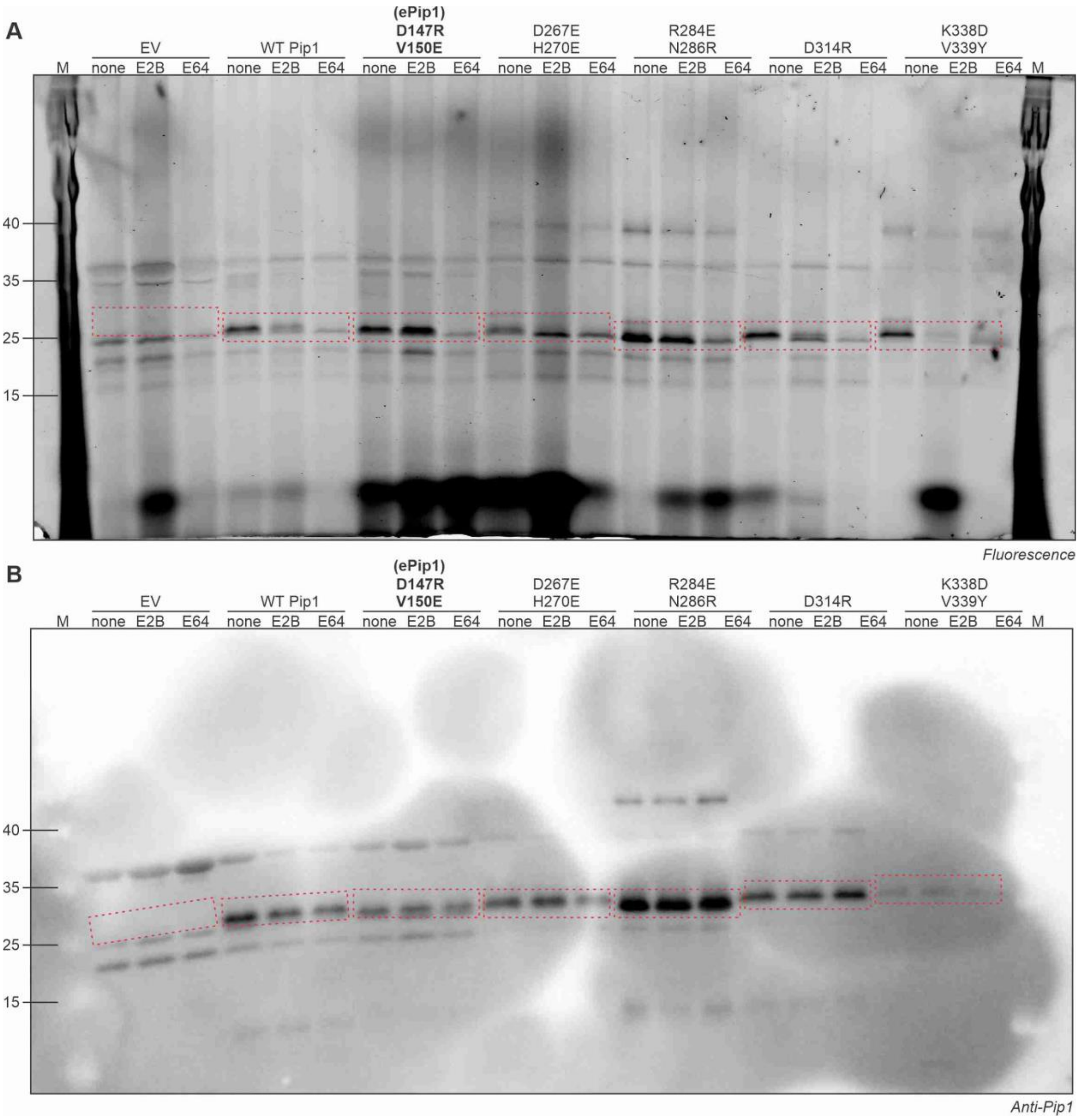

Figure S2 Uncropped gel and blot used for Fig1B.
Apoplastic fluids isolated from agroinfiltrated leaves transiently expressing (mutant) Pip1 were pre-incubated for 30 min with and without 100 µM E-64 or 3 µM EpiC2B, and then labelled for 3 hours with 0.2 µM TK011. Samples were separated on SDS-PAGE gels, scanned for fluorescence (top) and then transferred onto a membrane for an α-Pip1 western blot (bottom).

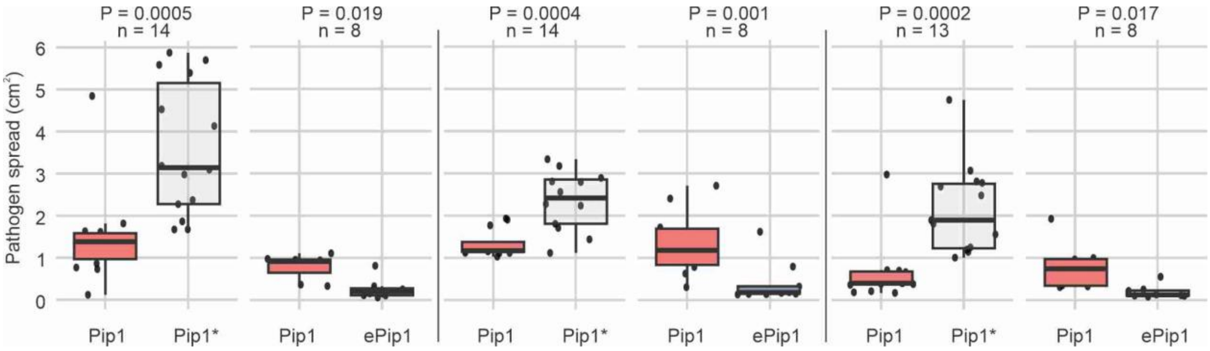

Figure S3 Overexpression of ePip1 in *N. benthamiana* restricts *P.infestans* spread.

Three independent replicates of the experiment are shown. Data of the second replicate was used in the main figure. P values correspond to paired *t*-test for n=x biological replicates.
